# Low Serum Magnesium Level Is Associated with Microalbuminuria in Chinese Diabetic Patients

**DOI:** 10.1155/2013/580685

**Published:** 2013-08-26

**Authors:** Baihui Xu, Jichao Sun, Xinru Deng, Xiaolin Huang, Wanwan Sun, Yu Xu, Min Xu, Jieli Lu, Yufang Bi

**Affiliations:** ^1^Key Laboratory for Endocrine and Metabolic Diseases of Ministry of Health, Rui-Jin Hospital, Shanghai Jiao-Tong University School of Medicine, E-Institute of Shanghai Universities, Shanghai 200025, China; ^2^Shanghai Clinical Center for Endocrine and Metabolic Diseases, Shanghai Institute of Endocrine and Metabolic Diseases, Department of Endocrinology and Metabolism, Rui-Jin Hospital, Shanghai Jiao-Tong University School of Medicine, Shanghai 200025, China

## Abstract

Whether serum magnesium deficiency is independently associated with the prevalence of microalbuminuria is still unclear. The objective of the present study was to elucidate the association between serum magnesium and microalbuminuria in diabetic patients. A cross-sectional study was conducted in 1829 diabetic subjects (aged ≥ 40 years) from Shanghai, China. Subjects were divided into three groups according to serum magnesium tertiles. A first-voided early-morning spot urine sample was obtained for urinary albumin-creatinine ratio (UACR) measurement. Microalbuminuria was defined as 30 mg/g ≤ UACR < 300 mg/g. Overall, 208 (11.37%) of the study population had microalbuminuria, with similar proportions in both genders (*P* = 0.44). The prevalence of microalbuminuria in tertile 1 of serum magnesium was higher than the prevalence in tertile 2 and tertile 3 (15.98%, 9.72%, and 8.46%, resp.; *P* for trend <0.0001). After adjustment for age, sex, BMI, blood pressure, lipidaemic profile, HbA1c, eGFR, history of cardiovascular disease, HOMA-IR, antihypertensive and antidiabetic medication, and diabetes duration, we found that, compared with the subjects in tertile 3 of serum magnesium, those in tertile 1 had 1.85 times more likeliness to have microalbuminuria. We concluded that low serum magnesium level was significantly associated with the prevalence of microalbuminuria in middle-aged and elderly Chinese.

## 1. Introduction 

Magnesium (Mg) is the fourth most abundant cation in the human body and is a critical cofactor in many enzymatic reactions [1, 2]. It plays an important role in many fundamental biological processes. Mg depletion is a common feature in diabetic patients [3, 4]. An Australian study demonstrated that hypomagnesaemia was 10.51-fold more common between patients with new diabetes and 8.63-fold more common between patients with known diabetes as compared with control subjects without diabetes [3]. In another large cohort of young American adults participating in the Coronary Artery Risk Development in Young Adults (CARDIA) study, it was shown that Mg intake was inversely longitudinally associated with the incidence of diabetes [4]. 

Microalbuminuria was first reported in diabetic patients by Viberti et al. in 1982 [5]. It has been shown to be associated with increased risk of cardiovascular morbidity and mortality in diabetic patients [6]. Furthermore, the presence of microalbuminuria is generally associated with a poorer glycometabolic control and a higher prevalence of chronic complications including diabetic retinopathy, peripheral vascular disease, and diabetic neuropathy [7].

The association between microalbuminuria and Mg depletion is a controversial issue. A previous report showed that high doses of Mg reduce microalbuminuria in traumatic critically ill patients at 36 hour, after infusion [8]. Conversely, there were no significant differences between patients with hypomagnesemia and normal subjects with respect to microalbuminuria [9]. Therefore, the aim of the present study was to evaluate the association between serum Mg and microalbuminuria in diabetic patients in China.

## 2. Materials and Methods

### 2.1. Research Design and Subjects

This community-based cross-sectional study was conducted in Jiading district, Shanghai, China, from March to August, 2010. In brief, 10375 subjects, aged 40 years or above, were enrolled to participate in the survey. Among those subjects, there were 1872 diabetic patients, those with fasting plasma glucose (FPG) ≥ 7.0 mmol/L and/or 2 h plasma glucose (2 h-PG) ≥ 11.1 mmol/L or with a history of diabetes. The diagnosis of diabetes was defined according to the 1999 World Health Organization criteria [10]. Microalbuminuria was defined as 30 mg/g ≤ urinary albumin-creatinine ratio (UACR) < 300 mg/g [11]. For analysis, we excluded subjects who had missing data on serum Mg or urine albumin or urine creatinine (*n* = 5), those who had urinary tract infection, glomerulonephritis, nephritic syndrome, or kidney cancer (*n* = 17), and those who had UACR ≥ 300 mg/g (*n* = 21). Finally, a total of 1829 diabetic subjects (775 males and 1054 females) were included in the analysis. 

This study was conducted with the approval of the institutional review board of Ruijin Hospital affiliated to Shanghai Jiao-Tong University School of Medicine. All participants provided informed consent. 

### 2.2. Clinical Data Collection and Biochemical Measurements

The information about demographic characteristics, lifestyle, the history of chronic diseases, and current use of medication, including antihypertensive drugs and antidiabetic drugs, were obtained by a standard interview questionnaire. Current smokers or drinkers were defined as subjects who smoked cigarettes or consumed alcohol regularly in the past 6 months, while subjects who never or formerly smoked cigarettes or consumed alcohol were defined as noncurrent smokers or noncurrent drinkers.

Blood pressure was measured at the nondominant arm three times consecutively at 1 min intervals after subjects had rested for at least 5 min in a sitting position, using an automated electronic device (OMRON Model HEM-752; Omron, Dalian, China). The average of the three measurements was used in the analysis. Subjects with systolic blood pressure (SBP) ≥ 140 mmHg and/or diastolic blood pressure (DBP) ≥ 90 mmHg or taking antihypertensive drugs were defined as having hypertension [12]. Body height and body weight were recorded to the nearest 0.1 cm and 0.1 kg while participants were wearing light indoor clothing without shoes. Body mass index (BMI) was calculated as body weight divided by squared body height (kg/m^2^). 

After at least 10 hours of overnight fasting, venous blood samples were collected for the measurements of serum insulin, blood glucose, lipid profile, serum creatine and glycated hemoglobin A1c (HbA1c). Blood glucose was measured with the use of the glucose oxidase method on an autoanalyser (Modular P800, Roche, Basel, Switzerland). Fasting serum insulin, serum creatinine, Mg, triglycerides (TG), total cholesterol (TC), high-density lipoprotein cholesterol (HDL-c), and low-density lipoprotein cholesterol (LDL-c) were measured by an autoanalyser (Modular E170, Roche, Basel, Switzerland). HbA1c was assessed by high-performance liquid chromatography (HPLC, BIO-RAD D-10, USA). The insulin resistance index (homeostasis model assessment of insulin resistance, HOMA-IR) was calculated as fasting insulin (*μ*IU/mL) × fasting glucose (mmol/L)/22.5 [13]. GFR was estimated based on serum creatinine concentration using the modification of diet in renal disease (MDRD) formula: eGFR = [186 × serum creatinine (umol/L) × 0.0113]^−1.154^ × age^−0.203^ × (0.742 for women) [14].

A first-morning spot urine sample was obtained at the survey center. Women experiencing menstruation on the survey day were not included in the present study. Urine albumin and creatinine were measured by immunoturbidimetric method (Beijing Atom High-Tech, Beijing, China) and the Jaffe's kinetic method on an automatic analyser (Hitachi 7600-020, Tokyo, Japan), respectively. The UACR in mg/g was calculated as urine albumin concentration divided by urine creatinine concentration.

### 2.3. Statistical Analysis

Participants were divided into tertitles according to serum Mg concentration as tertile 1: Mg < 0.86 mmol/L, tertile 2: 0.86 mmol/L ≤ Mg < 0.92 mmol/L, and tertile 3: Mg ≥ 0.92 mmol/L. Baseline characteristics of subjects were calculated as mean and standard deviation (SD), median and interquartile range, or percentage. Trends in means and proportions were tested using linear regression and *χ* ^2^ tests, respectively. HbA1c, HOMA-IR, TG, and UACR were logarithmically transformed before analysis due to a nonnormal distribution.

Logistic regression was used to evaluate the association between serum Mg and the prevalence of microalbuminuria. Model 1 was unadjusted. In Model 2, we adjusted for age, sex, and BMI. In Model 3, we further adjusted for SBP, DBP, LDL-c, HDL-c, TC, TG, HbA1c and history of cardiovascular disease. In Model 4, we additionally adjusted for HOMA-IR, eGFR, antihypertensive drugs, antidiabetic drugs, and diabetes duration. Relationship between serum Mg and the prevalence of microalbuminuria was also explored in stratified analysis. The factors associated with serum Mg levels or UACR were considered as the strata factors. Odds ratios were calculated for each tertile decline of serum Mg levels in subgroups of the strata variables.

All analysis were performed with SAS (version 9.3; SAS Institute, Cary, NC, USA). *P* < 0.05 was considered statistically significant.

## 3. Results

### 3.1. Characteristics of the Study Population

Demographic and clinical characteristics and biochemical measurements of 1829 subjects according to tertiles of serum Mg are shown in Table 1. Compared with subjects in the higher serum Mg group, those with lower serum Mg level were more likely to be females and had higher prevalence of antidiabetic drugs use, higher level of FPG, 2 h-PG, HbA1c, HOMA-IR, UACR, and eGFR, and lower level of LDL-c, TC, and serum creatinine (all *P* for trend <0.05). However, age, BMI, SBP, DBP, HDL-c, TG, antihypertensive drugs use, smoking status, and drinking status were not statistically different among the three groups.

### 3.2. Prevalence of Microalbuminuria in Different Serum Mg Levels

**Figure 1:**
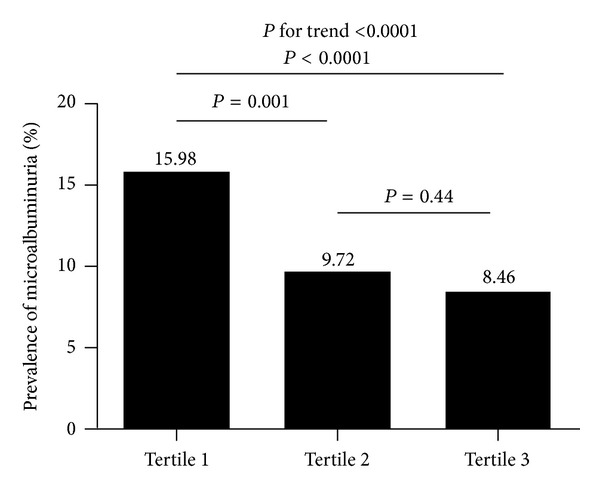
Prevalence of microalbuminuria by serum Mg tertiles (the serum Mg tertiles cutoff points were tertile 1: Mg < 0.86 mmol/L, and tertile 2: 0.86 mmol/L ≤ Mg < 0.92 mmol/L, tertile 3: Mg ≥ 0.92 mmol/L).

Overall, 208 (11.37%) of the study population had microalbuminuria, with similar proportions in males and females (10.71% versus 11.86%; *P* = 0.44). As shown in Figure 1, across the serum Mg tertiles, the prevalence of microalbuminuria was 15.98%, 9.72%, and 8.46%, respectively (*P* for trend <0.0001). Strikingly, a significant increase was observed in tertile 1 compared with tertile 2 (*P* = 0.001) and tertile 3 (*P* < 0.0001), respectively. However, the difference of the prevalence of microalbuminuria between tertile 2 and tertile 3 was not statistically significant (*P* = 0.44).

### 3.3. Serum Mg in relation to Microalbuminuria

As shown in Table 2, declined serum Mg was strongly associated with an increased prevalence of microalbuminuria in both univariate and multivariate analyses. In the univariate model, the presence of microalbuminuria was significantly more frequent among the participants in tertile 1 than those in tertile 3 (OR = 2.06, 95% CI: 1.44–2.95). After further adjustment for age, sex, BMI, SBP, DBP, LDL-c, HDL-c, TC, TG, HbA1c, eGFR, history of cardiovascular disease, HOMA-IR, antihypertensive drugs, antidiabetic drugs and diabetes duration (Model 4), the ORs for microalbuminuria among patients in tertile 1, and 2 in comparison with tertile 3 were 1.85 (95% CI: 1.26–2.72) and 1.11 (95% CI: 0.74–1.67), respectively.

Multivariate-adjusted OR for microalbuminuria with each tertile decrease of serum Mg in different subgroups is shown in Table 3. The associations between serum Mg and the prevalence of microalbuminuria were not subgroup consistent. Significant associations were detected in males, subjects with higher HbA1c (≥6.5%), subjects with or without hypertension, and subjects with shorter duration of diabetes (<10 year).

## 4. Discussion

In this cross-sectional study, we found a significant inverse association between serum Mg concentration and the prevalence of microalbuminuria in middle-aged or elderly Chinese. Moreover, the relationship was independent of other confounding factors.

Our findings are generally consistent with the results from some previous studies. For instance, Corsonello et al. demonstrated that diabetic patients with microalbuminuria or overt proteinuria showed a significant decrease in serum Mg compared with normoalbuminuria group [7]. It has been reported that, compared with type 1 diabetic patients with normoalbuminuria, a significant reduction in serum Mg levels has been found in type 1 diabetic patients with microalbuminuria or clinical proteinuria [15]. Evidence also suggested that non-insulin-dependent diabetic patients with hypomagnesemia showed an increased urinary albumin excretion rate with respect to normomagnesemic diabetic patients [16].

In contrast, other studies did not find any significant associations between serum Mg and microalbuminuria. A previous study on type 1 diabetic patients has shown that there was no association between microalbuminuria and serum total Mg concentration [17]. In addition, a cross-sectional study in Brazil did not find any significant difference in microalbuminuria between type 2 diabetic patients with plasma Mg <0.75 mmol/L and type 2 diabetic patients with plasma Mg ≥0.75 mmol/L [9].

The possible reasons for the inconsistence of our results with the above previous studies are shown as follows. (1) The JACC study from Japan demonstrated that dietary magnesium intake was associated with reduced mortality from cardiovascular disease [18]. On the other hand, microalbuminuria is considered as an independent predictor of cardiovascular disease [19]. Thus, different habits of food intake from different countries may affect the association between serum Mg concentration and microalbuminuria. (2) The sample size of above studies were too small to demonstrate the relationship between serum Mg and microalbuminuria.

One of the potential pathophysiological mechanisms linking serum Mg to microalbuminuria is amplification of insulin resistance. It was said that low serum Mg plays an important role in pathogenesis of insulin resistance. Mg can function as a mild, natural calcium antagonist. So the level of intracellular calcium is increased in Mg-deficiency subjects. This increased intracellular calcium may compromise the insulin responsiveness of adipocytes and skeletal muscles leading to the development of insulin resistance [20]. Another study has also found that insulin deficiency or insulin resistance can affect the tubular absorption of Mg, leading to hypomagnesemia in diabetic subjects [21]. We speculated that a vicious circle formed by mutual influence between insulin resistance and hypomagnesemia results in aggravation of insulin resistance which can increase the risk of microalbuminuria [22]. 

Oxidative stress is becoming increasingly recognized as an important causative factor for microalbuminuria [23]. Mg has been reported to possess antioxidant property [24]. Hence, oxidative stress may be one of the mechanisms that underlie the association between low serum Mg and microalbuminuria. Study has also shown that Mg intake and serum Mg concentration were inversely associated with systemic inflammation markers [4], which also play an crucial role in the pathogenesis of microalbuminuria [8].

Our study adds evidence to the association between low serum Mg and microalbuminuria. However, there are several limitations that require consideration. First, lack of dietary Mg measurement is one limitation of the present study which may impede us to determine the effect of low dietary Mg intake on serum Mg level and risk of prevalent microalbuminuria. Second, no causal inference can be drawn due to the cross-sectional design of the current study. Further prospective studies are needed to illustrate the precise relationship between Mg depletion and incident of microalbuminuria. Third, UACR levels were determined by a single measurement and may not be accurately representative of the status of study subjects. 

## 5. Conclusions 

In summary, serum Mg was inversely associated with the prevalence of microalbuminuria. Further large-scale clinical trials are needed to be carried out to determine whether correction of Mg deficiency, through medications or dietary intake, could be effective to reduce the incidence of microalbuminuria and elucidate the mechanisms underlying the association between serum Mg and microalbuminuria.

## Figures and Tables

**Table 1:** General characteristics of the study population.

Variables	Serum magnesium levels (mmol/L)			*P* for trend
	Tertile 1 Mg < 0.86	Tertile 2 0.86 ≤ Mg < 0.92	Tertile 3 Mg ≥ 0.92	
*N*	607	607	615	
Age (year)	61.11 ± 10.01	61.53 ± 9.18	61.98 ± 9.98	0.10
Female (%)	62.11	57.00	53.82	0.003
BMI (kg/m^2^)	26.31 ± 3.50	26.24 ± 3.40	26.08 ± 3.50	0.24
Current smoking (%)	19.28	20.59	19.84	0.81
Current drinking (%)	9.72	10.71	9.92	0.91
Antidiabetic drugs (%)	47.12	40.53	35.45	<0.0001
Antihypertensive drugs (%)	41.19	41.19	46.18	0.08
FPG (mmol/L)	8.34 ± 2.97	7.35 ± 2.18	7.02 ± 1.89	<0.0001
2h-PG (mmol/L)	16.60 ± 5.86	15.35 ± 4.66	14.24 ± 4.33	<0.0001
HbA1c (%)	6.9 (6.2–8.3)	6.6 (6.0–7.4)	6.3 (5.8–7.0)	<0.0001
HOMA-IR	2.93 (1.91–4.92)	2.86 (1.77–4.35)	2.67 (1.63–4.12)	0.002
SBP (mmHg)	149.60 ± 19.95	149.53 ± 20.23	148.09 ± 18.57	0.18
DBP (mmHg)	84.40 ± 10.30	83.92 ± 10.58	83.64 ± 9.90	0.20
LDL-c (mmol/L)	3.23 ± 0.94	3.31 ± 0.92	3.34 ± 0.91	0.03
HDL-c (mmol/L)	1.28 ± 0.30	1.27 ± 0.31	1.26 ± 0.30	0.20
TC (mmol/L)	5.44 ± 1.12	5.49 ± 1.09	5.58 ± 1.08	0.02
TG (mmol/L)	1.60 (1.19–2.37)	1.67 (1.19–2.23)	1.74 (1.22–2.44)	0.11
UACR (mg/g)	7.68 (3.94–17.94)	6.15 (3.43–13.58)	5.74 (3.00–12.72)	<0.0001
Serum creatinine (umol/L)	58.85 ± 14.95	61.15 ± 14.37	64.01 ± 16.98	<0.0001
eGFR (mL/min per 1.73 m^2^)	114.90 ± 27.58	110.16 ± 23.05	105.85 ± 22.73	<0.0001

Data are means ± SD, medians (interquartile range), or percentages of subjects.

**Table 2:** Association between serum Mg levels and the prevalence of microalbuminuria.

	Serum Mg levels		
	Tertile 3	Tertile 2	Tertile 1
Model 1	1.00 (reference)	1.17 (0.79–1.72)	2.06 (1.44–2.95)
Model 2	1.00 (reference)	1.17 (0.79–1.73)	2.09 (1.45–3.00)
Model 3	1.00 (reference)	1.06 (0.71–1.59)	1.74 (1.19–2.54)
Model 4	1.00 (reference)	1.11 (0.74–1.67)	1.85 (1.26–2.72)

Model 1: unadjusted.

Model 2: adjusted for age, sex, and BMI.

Model 3: adjusted for age, sex, BMI, SBP, DBP, LDL-c, HDL-c, TC, TG, HbA1c, and history of cardiovascular disease.

Model 4: adjusted for age, sex, BMI, SBP, DBP, LDL-c, HDL-c, TC, TG, HbA1c, eGFR, history of cardiovascular disease, HOMA-IR, antihypertensive drugs, antidiabetic drugs, and diabetes duration.

**Table 3:** The risk of microalbuminuria with each tertile decline of serum Mg in different subgroups of diabetic patients.

	Number of microalbuminuria/number of subjects	Multivariate^a^	
		OR (95% CI)	*P*
Sex			
Male	83/775	1.71 (1.22–2.38)	0.002
Female	125/1054	1.20 (0.93–1.53)	0.16
HbA1c			
6.5%	81/861	1.11 (0.82–1.51)	0.48
≥6.5%	127/968	1.69 (1.30–2.20)	<0.0001
Hypertension			
Absence	24/392	1.85 (1.00–3.40)	0.05
Presence	184/1436	1.37 (1.11–1.69)	0.003
Duration of diabetes			
<10 year	187/1684	1.42 (1.16–1.74)	0.0008
≥10 year	21/145	0.98 (0.48–1.98)	0.95

^a^adjusted for age, sex, BMI, SBP, DBP, LDL-c, HDL-c, TC, TG, HbA1c, eGFR, history of cardiovascular disease, HOMA-IR, antihypertensive drugs, antidiabetic drugs, and diabetes duration (except for the strata variables).
